# 改性苯乙烯-马来酸酐共聚物色谱固定相用于磷脂分离分析

**DOI:** 10.3724/SP.J.1123.2023.02011

**Published:** 2023-10-08

**Authors:** Yangyang NIE, Guantao YANG, Haiyan WANG, Xiaoqiang QIAO

**Affiliations:** 1.河北大学药学院, 河北 保定 071002; 1. School of Pharmacy, Hebei University, Baoding 071002, China; 2.保定市清苑区人民医院, 河北 保定 071100; 2. Baoding Qingyuan People’s Hospital, Baoding 071100, China

**Keywords:** 苯乙烯-马来酸酐共聚物, 色谱固定相, 磷脂, 点击反应, 自由基聚合, styrene-maleic anhydride copolymer, chromatographic stationary phase, phospholipid, “click” reaction, free radical polymerization

## Abstract

磷脂是重要的信号分子,磷脂的代谢与多种疾病密切相关。因此,开展磷脂的分离分析研究至关重要。苯乙烯-马来酸酐共聚物(SMA)作为一种新型两亲性交替共聚物可以插入生物膜的磷脂双分子层中,形成以膜蛋白质为中心的脂质纳米盘,对膜蛋白质和磷脂具有良好的增溶作用。本文基于“点击”反应和自由基聚合反应,将对磷脂具有良好增溶性能的SMA接枝到硅胶表面,然后以蛋氨酸甲酯盐酸盐(MME·HCl)为开环试剂,通过亲核开环反应对SMA进行修饰,制备了新型的改性SMA修饰色谱固定相(Sil-SMA-MME)。结合高效液相色谱-紫外检测法,利用酰胺类和核苷/核酸碱基类以及苯酚类3类小分子物质对填充Sil-SMA-MME色谱柱的保留机制和分离性能进行了系统评价,Sil-SMA-MME色谱柱具有典型的亲水作用保留机制,其柱效最高可达90900 N/m,并显示了良好的分离选择性。进一步结合高效液相色谱-蒸发光散射检测法,考察了Sil-SMA-MME色谱柱对磷脂样品的分离性能。二棕榈酰磷脂酰丝氨酸钠(DPPS)、二油酰磷脂酰胆碱(DOPC)、二棕榈酰磷脂酰乙醇胺(DPPE)和4种磷脂酰胆碱(PC)类标准品——溶血卵磷脂(LysoPC)、二肉豆蔻酰磷脂酰胆碱(DMPC)、二硬脂酰磷脂酰胆碱(DSPC)、二棕榈酰磷脂酰胆碱(DPPC)均可实现基线分离,并且成功地实现了南极磷虾油和人血清磷脂提取物的分离分析。以上结果表明,所制备的Sil-SMA-MME色谱柱在磷脂类物质分离分析中具有良好的应用潜力。

## Abstract (en)

Phospholipids are important signaling molecules, and their metabolism is closely related to various diseases, such as neurodegenerative diseases and cancers. Phospholipids are typically characterized with extreme complexity and structural diversity. For example, phospholipids present in many different forms, such as *sn* position isomers, double-bond position isomers, double-bond stereochemical isomers, and enantiomers. Therefore, further research on novel separation and analytical techniques for phospholipids is of great importance. As an amphiphilic alternating copolymer, styrene-maleic anhydride copolymer (SMA) can be inserted into the phospholipid bilayer of biofilms to form lipid nanodisks with membrane proteins as the centers, thereby solubilizing membrane proteins and phospholipids. Thus, the introduction of SMA into a chromatographic stationary phase can potentially improve the separation and analysis of phospholipids. In this paper, SMA was successfully grafted onto the surface of silica gel via the “click” reaction and free radical polymerization. After further ring-opening modification of SMA with methyl methionine hydrochloride (MME·HCl), a novel SMA-modified stationary phase material (Sil-SMA-MME) was fabricated. The Sil-SMA-MME stationary phase was characterized using thermogravimetric analysis and Fourier transform infrared spectroscopy (FT-IR), and the results indicated the successful fabrication of the target material. The retention mechanism of the packed Sil-SMA-MME chromatographic column was investigated using hydrophilic nucleosides and nucleic acid bases via high performance liquid chromatography (HPLC) and UV detection. According to the retention characteristics of the nucleosides and nucleic acid bases in different mobile phases, the Sil-SMA-MME chromatographic column exhibited a typical hydrophilic-interaction-based retention mechanism, similar to that of a commercially available amino (SiO_2_-NH_2_) column. The separation performance of the Sil-SMA-MME column was evaluated using three types of small-molecule substances, including amides, nucleoside/nucleic acid bases, and phenols. Cyanoacetamide, 2-iodoacetamide, benzamide, *p*-aminobenzamide, and nicotinamide were used to evaluate the chromatographic performance of the developed Sil-SMA-MME column. When acetonitrile-H_2_O (96∶4, v/v) was used as the mobile phase, the five compounds exhibited good peak shapes and could be baseline-separated within 8 min. The highest column efficiency achieved was 90900 N/m. By contrast, under the same chromatographic conditions, the test substances were not separated effectively on the SiO_2_-NH_2_ column. Regardless of the mobile phase ratio, the peaks of benzamide and 2-iodoacetamide overlapped. These results demonstrate that the developed Sil-SMA-MME column has good separation selectivity. The separation performance of the Sil-SMA-MME column for phospholipid samples was also investigated by HPLC and evaporative light scattering detection (ELSD) to explore its feasibility for phospholipid separation and analysis. Different phospholipid standards were used to evaluate the separation performance of the column. Under certain mobile phase conditions, baseline separation could be achieved for dipalmityl phosphatidyl serine sodium (DPPS), diolyl phosphatidyl choline (DOPC), and dipalmityl phosphatidyl ethanolamine (DPPE), as well as four phosphatidyl choline (PC) standards, namely, lysophosphatidylcholine (LysoPC), dimyristoyl phosphatidyl choline (DMPC), distearyl phosphatidyl choline (DSPC), and dipalmitoyl phosphatidyl choline (DPPC). The separation potential of the developed Sil-SMA-MME column was further evaluated by separating and analyzing phospholipid extracts from Antarctic krill oil and human serum. The results showed that the developed Sil-SMA-MME column has good potential for phospholipid separation and analysis.

细胞或者组织的脂质组成变化与多种疾病的发生息息相关^[1,2]^。近年来,人们逐渐认识到脂质的重要性,与脂质相关的生物学功能及其相关的分析技术研究越来越多地受到研究人员的关注^[3⇓⇓⇓-7]^。磷脂是重要的脂类物质之一,是生物膜的主要成分,在生命体中起着重要的结构、代谢和功能作用^[8⇓-10]^。此外,磷脂还参与细胞死亡和细胞信号转导^[11]^,并且与各种神经退行性疾病和肿瘤息息相关^[12⇓-14]^。由于其生化和临床的重要性,发展快速可靠的磷脂分离分析方法对于识别和定量生物样本中的复杂磷脂分子具有重要意义。

磷脂分子结构复杂、种类繁多且含量各异,以最常见的磷脂酰胆碱(PC)为例,PC的主要异构体形式包括旋光异构、C=C位置异构、C=C顺反异构和*sn*位置异构,这些异构形式可以延伸出数以万计的异构体。因此,磷脂分子的极端复杂性使其分离分析面临着很大的挑战性。近年来,磷脂类物质的分离分析方法发展迅速,其中,高效液相色谱-质谱(HPLC-MS)技术成为磷脂分离、鉴定和定量的重要技术^[15]^。例如,Ansar等^[16]^基于HPLC-MS法分离和定量了Visudyne脂质体配方中的磷脂及其相应的降解产物,该方法能够分离磷脂的结构异构体和顺反异构体。Visudyne的主要脂质降解产物包括溶血磷脂酰胆碱、少数饱和/不饱和溶血磷脂酰甘油以及游离的脂肪酸,每种降解产物含量均小于磷脂总量的1%(质量分数)。Sun等^[17]^开发了基于超高效液相色谱-四极杆飞行时间质谱的方法来分析人乳、牛奶和羊奶中的磷脂类别与分子种类,共鉴定出11个磷脂类,包含229种磷脂分子。目前,磷脂分离常用的HPLC色谱柱包括二醇柱、C18柱、氨基柱等^[18⇓⇓⇓⇓-23]^。近年来,多种新型的色谱固定相被研制出来以应对复杂磷脂样品的分离需求。例如,Liu等^[24]^以烯丙基三辛基溴化膦为单体,设计合成了新型聚膦离子液体键合硅胶色谱固定相(PIL@SiO_2_),可同时实现磷脂类和磷脂分子的分离,并成功应用于大豆卵磷脂中磷脂的分离分析。李新庭等^[25]^以溴化1-乙烯基-3-十二烷基咪唑(VDI)为单体,制备了VDI键合色谱固定相(Sil-VDI),该固定相具有反相/离子交换混合模式保留机制和良好的分离性能。作者进一步将其用于肺癌细胞和鸡蛋黄磷脂样品提取物的分离分析,Sil-VDI色谱柱对2种磷脂样品均展示了良好的分离选择性。然而,为了改善复杂磷脂样品的分离分析性能,研制新型磷脂色谱分离固定相仍然是目前研究的热点之一。

苯乙烯-马来酸酐共聚物(SMA)是一类新型两亲性聚合物,在磷脂和膜蛋白质增溶方面得到了广泛的应用^[26,27]^, SMA对膜蛋白质增溶的原理是其可以和膜蛋白质周围的磷脂双分子层发生亲水、疏水相互作用和静电相互作用,从而插入其中形成“纳米圆盘”^[28,29]^,实现对膜蛋白质的增溶。基于SMA与磷脂双分子层的强相互作用,本文将SMA引入硅胶色谱固定相的制备,并采用蛋氨酸甲酯盐酸盐(MME·HCl)对马来酸酐基团进行开环修饰,合成了新型的改性SMA修饰色谱固定相(Sil-SMA-MME),并将该固定相用于南极磷虾油和人血清磷脂提取物的分离分析。

## 1 实验部分

### 1.1 仪器与试剂

Nicoleti S10傅里叶变换红外光谱仪(美国Nicolet公司); GLK型装柱机、不锈钢色谱柱管(250 mm×4.6 mm)、GALAK型氨基色谱柱(SiO_2_-NH_2_)(250 mm×4.6 mm)(无锡加莱克色谱科技有限公司); P230Ⅱ高效液相色谱仪(大连依利特分析仪器有限公司); ELSD6000蒸发光散射检测器(ELSD)(美国奥泰科技有限公司); STA449C热重分析仪(德国耐驰公司); IKA RCT basic磁力搅拌器(艾卡仪器设备有限公司); SB-100DT超声波清洗机(宁波新芝生物科技股份有限公司)。

球形多孔硅胶(粒径5 μm,孔径12 nm,日本Daisogel公司); (3-巯丙基)三乙氧基硅烷(MPS,纯度96%)、MME·HCl(纯度98%)、苯乙烯(纯度99%)、马来酸酐(纯度99%)、偶氮二异丁腈(AIBN,纯度99%)、对甲苯磺酸(TsOH,纯度99%)(北京伊诺凯科技有限公司); *N*,*N*-二甲基甲酰胺(DMF)、甲基叔丁基醚(MTBE)(天津市科密欧化学试剂有限公司);二棕榈酰磷脂酰胆碱(DPPC,纯度98%)、二硬酯酰磷脂酰胆碱(DSPC,纯度95%)、二肉豆蔻酰磷脂酰胆碱(DMPC,纯度99%)、二棕榈酰磷脂酰乙醇胺(DPPE,纯度97%)(北京百灵威科技有限公司);溶血卵磷脂(LysoPC,纯度97%,天津阿尔塔科技有限公司);二油酰磷脂酰胆碱(DOPC,纯度97%,梯希爱上海化成工业发展有限公司);二棕榈酰磷脂酰丝氨酸钠(DPPS,纯度95%,艾美捷科技有限公司);甲醇(MeOH)和乙腈(ACN)(HPLC级,天津赛孚瑞科技有限公司)。

### 1.2 Sil-SMA-MME固定相的制备

试剂预处理 称取硅球2.5 g,放入真空干燥箱中,于100 ℃烘干24 h备用。

巯基化硅球(Sil-SH)的制备 取干燥后的硅球,将其置于250 mL圆底烧瓶中,随后加入50 mL无水甲苯使硅球在溶剂中混悬,之后加入5.8 g MPS,在氮气氛围下于110 ℃搅拌反应12 h。反应结束后,离心除去溶液,沉淀的硅球用甲醇洗涤5次,在80 ℃下真空干燥24 h,得到Sil-SH。

苯乙烯-马来酸酐共聚物接枝硅球(Sil-SMA)的制备 称取14.7 g (150 mmol)马来酸酐,置于500 mL圆底烧瓶中,加入50 mL DMF超声使其充分溶解,加入上一步得到的Sil-SH,继续加入31.3 g (300 mmol)苯乙烯后立即加入60 mL DMF振荡,使溶液充分混匀,再加入溶于10 mL DMF中的0.69 g AIBN,立即将溶液置于氮气氛围下,于80 ℃搅拌反应16 h。反应结束后,离心除去黏稠溶液,沉淀的硅球用DMF离心洗涤至上清液无色,再用甲醇洗涤2次,于70 ℃下真空干燥24 h,得到Sil-SMA。

Sil-SMA-MME固定相的制备 称取4.0 g (20 mmol) MME·HCl,置于250 mL圆底烧瓶中,加入30 mL DMF超声使其充分溶解,取上一步反应产物加入溶液中,再加入50 mL DMF振荡,使溶液充分混匀,称取TsOH 0.5 g,用10 mL DMF溶解后倒入烧瓶中,在氮气氛围下于85 ℃搅拌反应5 h。反应结束后,产物依次用DMF和甲醇各离心洗涤3次,在70 ℃下真空干燥24 h,得到最终的固定相材料。

以上合成步骤见图1。

**图 1:**
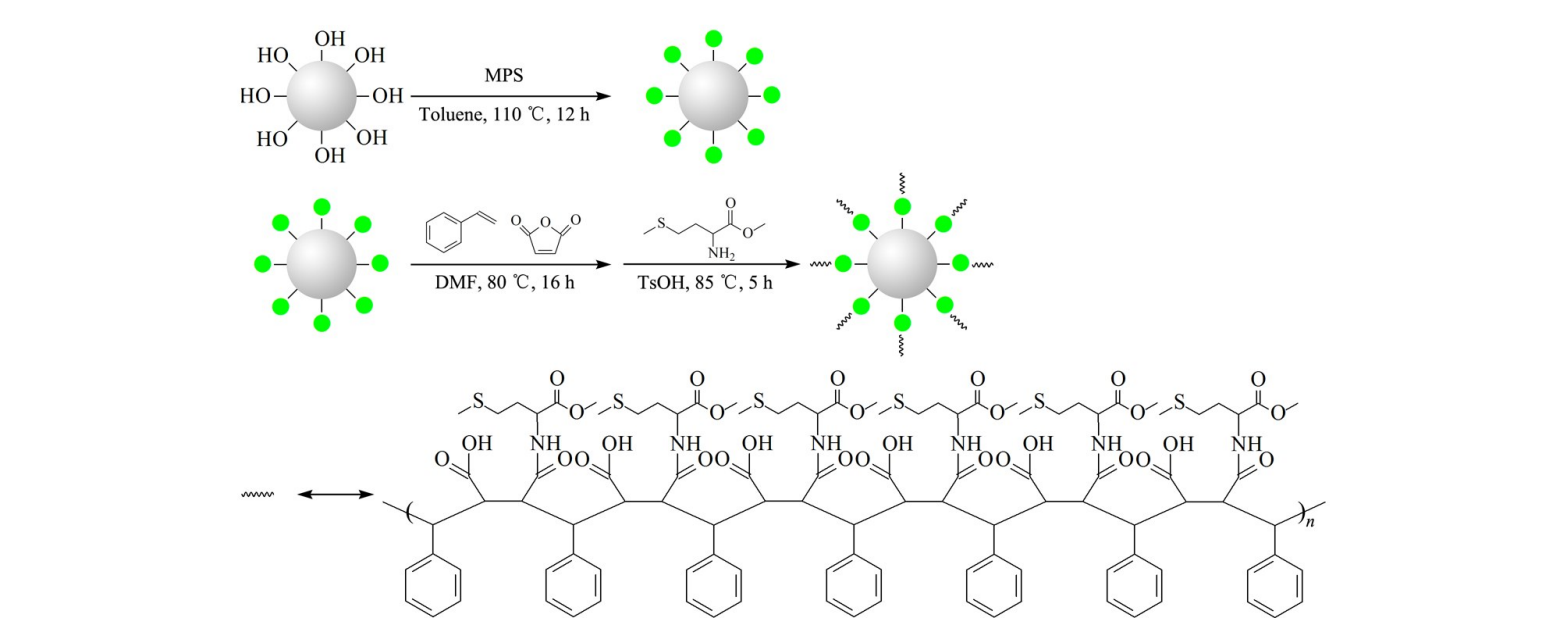
Sil-SMA-MME固定相制备示意图

### 1.3 色谱柱装填

采取高压匀浆法装填Sil-SMA-MME色谱柱。匀浆液为氯仿-环己醇(1∶4, v/v), 20 mL;顶替液为甲醇-异丙醇(1∶1, v/v)。称取Sil-SMA-MME 2.1 g,加入匀浆液搅拌使其分散均匀。将固定相材料的混悬液倒入匀浆管中,调节装柱机压力使其缓慢上升至35 MPa。待顶替液流出约100 mL后,装柱完成。使装柱机压力降为0 MPa,静置30 min后取下色谱柱,安装剩余的筛板和柱头,连接高效液相色谱泵,用乙腈冲洗色谱柱,备用。

### 1.4 样品配制

#### 1.4.1 色谱测试标准品的配制

本实验中所有小分子化合物样品均采用色谱纯乙腈配制并经过超声、离心、过膜后备用。酰胺类化合物中氰基乙酰胺质量浓度为80 μg/mL,其余小分子化合物质量浓度均控制在30~50 μg/mL。

#### 1.4.2 磷脂标准品与复杂磷脂样品的制备

DPPC、LysoPC、DOPC、DSPC和DMPC采用三氯甲烷-甲醇(1∶1, v/v)配制,其余磷脂类标准品采用三氯甲烷-甲醇(3∶1, v/v)配制。磷脂标准品质量浓度控制在0.5~1.0 mg/mL,所有样品均采用0.45 μm有机滤膜过滤,-20 ℃冷藏备用。

南极磷虾油磷脂提取:将磷虾油胶囊剪开,称取胶囊内容物100 mg,用20 mL正己烷-异丙醇(1∶1, v/v)溶解,所得样品超声5 min,用0.45 μm有机滤膜过滤,常温下放置备用。

人血清中磷脂提取物的制备:采用MTBE-MeOH体系,在200 μL人血清中加入400 μL MTBE和80 μL MeOH,涡旋30 s,以3000 r/min离心15 min,此时两相分离,界面出现白色沉淀,取上层有机相,用氮气吹干,并用1 mL二氯甲烷-甲醇(1∶1, v/v)复溶,用0.45 μm有机滤膜过滤后冷藏备用。

## 2 结果与讨论

### 2.1 结构表征

首先采用热重分析对Sil-SMA-MME固定相进行表征。如图2a所示,与SiO_2_相比,Sil-SH、Sil-SMA和Sil-SMA-MME的热损失逐渐增加,可验证Sil-SMA-MME固定相制备成功。进一步采用傅里叶变换红外光谱法对Sil-SMA-MME固定相进行表征。如图2b所示,在Sil-SMA的红外光谱图中出现了1495 cm^-1^和1455 cm^-1^处的苯环骨架伸缩振动吸收峰、700 cm^-1^处的苯环C-H面外弯曲振动吸收峰以及1830 cm^-1^和1735 cm^-1^处的马来酸酐不对称伸缩振动吸收峰和对称伸缩振动吸收峰,由此可以证实苯乙烯和马来酸酐已经成功接枝到硅球表面。与Sil-SMA相比,Sil-SMA-MME固定相红外光谱图中1735 cm^-1^处吸收峰变小,证明部分酸酐基团已与MME发生酰化反应,可进一步验证Sil-SMA-MME固定相制备成功。

**图 2:**
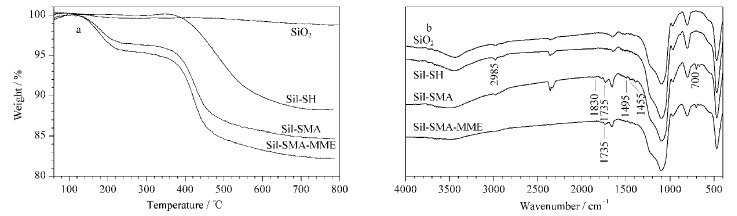
SiO_2_、Sil-SH、Sil-SMA和Sil-SMA-MME的(a)热重分析图和(b)红外光谱图

### 2.2 Sil-SMA-MME色谱柱的保留机制

Sil-SMA-MME固定相表面的酸酐基团经修饰后又引入羧酸基团,因此Sil-SMA-MME色谱柱可具有亲水保留特性。选取氰基乙酰胺、苯甲酰胺、2-碘乙酰胺、对氨基苯甲酰胺和烟酰胺对Sil-SMA-MME色谱柱的保留机制进行考察。从图3中可以看出,Sil-SMA-MME色谱柱在高比例乙腈的流动相系统中可以很好地保留5种酰胺类物质。当流动相中乙腈体积分数从95%下降到70%时,5种物质的保留逐渐减弱,表现出典型的亲水作用色谱保留机制。

**图 3:**
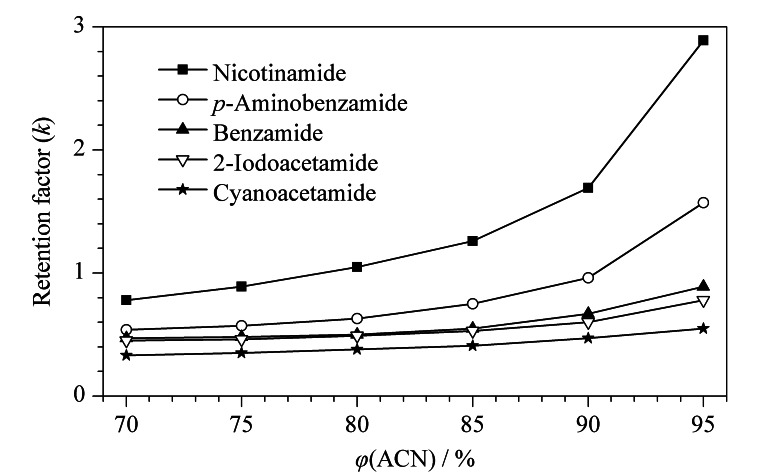
流动相中乙腈体积分数对亲水性酰胺类物质保留的影响

### 2.3 Sil-SMA-MME色谱柱的分离性能考察

#### 2.3.1 分离酰胺类化合物

选取5种酰胺类小分子物质对Sil-SMA-MME色谱柱的分离性能进行考察。图4为氰基乙酰胺、2-碘乙酰胺、苯甲酰胺、对氨基苯甲酰胺和烟酰胺的分离色谱图。以乙腈-水(96∶4, v/v)为流动相进行洗脱,5种物质在Sil-SMA-MME色谱柱中色谱峰形良好,其最高柱效达90900 N/m(图4a)。采用SiO_2_-NH_2_同样分离了上述5种物质,在相同的流动相条件下,SiO_2_-NH_2_色谱柱对于酰胺类物质的保留更弱,2-碘乙酰胺和苯甲酰胺的色谱峰完全重叠(图4b)。进一步调节流动相比例,使保留时间最长的烟酰胺与其在Sil-SMA-MME色谱柱上的保留时间一致,此时,2-碘乙酰胺和苯甲酰胺依旧无法分离(图4c)。这说明所制备的Sil-SMA-MME色谱柱可提供更高的分离选择性。

**图 4:**
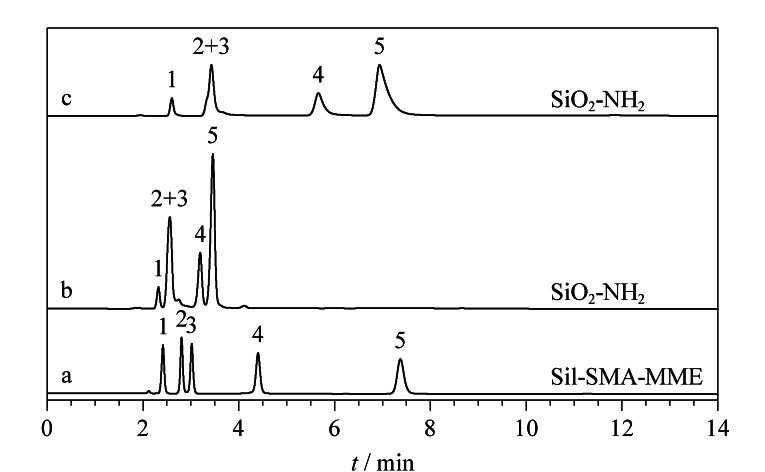
酰胺类物质在(a)Sil-SMA-MME色谱柱和(b, c)SiO_2_-NH_2_柱中的分离色谱图

#### 2.3.2 分离核苷/核酸碱基类化合物

进一步选用亲水性的核苷/核酸碱基类物质考察了Sil-SMA-MME色谱柱的分离性能。使用乙腈-水(94∶6, v/v)作为流动相对待测样品进行洗脱,在8 min内尿苷、尿嘧啶和肌苷可在Sil-SMA-MME色谱柱上达到基线分离,其最高柱效可达到79300 N/m(图5a)。同样采用SiO_2_-NH_2_对以上3种物质进行分离。在相同的流动相条件下,SiO_2_-NH_2_色谱柱对于核苷/核酸碱基类物质的保留更强,在15 min内只有尿嘧啶出峰(图5b)。进一步调节流动相中乙腈和水的比例,选用乙腈-水(60∶40, v/v)作为流动相,此时肌苷与其在Sil-SMA-MME色谱柱上的保留时间一致,但尿嘧啶和尿苷的分离度仅为1.62(图5c)。

**图 5:**
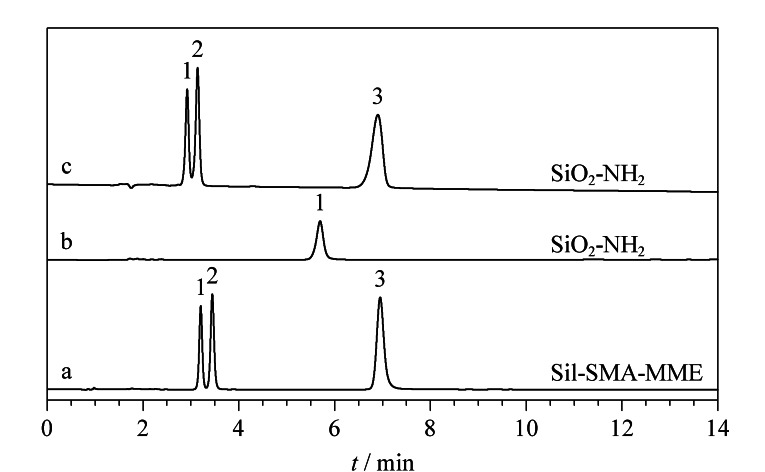
核苷/核酸碱基类物质在(a)Sil-SMA-MME色谱柱和(b, c)SiO_2_-NH_2_柱中的分离色谱图

#### 2.3.3 分离苯酚类化合物

选取了4种苯酚类小分子物质对Sil-SMA-MME色谱柱的分离性能进行考察。图6a为对乙酰氨基酚、对甲酚、对氯苯酚和4-叔辛基苯酚在Sil-SMA-MME色谱柱上的分离色谱图。以流动相乙腈-水(40∶60, v/v)进行洗脱,4种物质在6 min内可实现基线分离,其最高柱效可达到60000 N/m。采用SiO_2_-NH_2_对以上4种物质进行分离,在相同的流动相条件下,SiO_2_-NH_2_色谱柱对于苯酚类物质的保留更弱,其中对乙酰氨基酚和对甲酚的色谱峰完全重叠,对氯苯酚和4-叔辛基苯酚也无法达到基线分离(图6b)。调节流动相比例,使保留时间最长的4-叔辛基苯酚与其在Sil-SMA-MME色谱柱上保留时间一致,此时4种物质可以达到基线分离(图6c),但两种色谱柱表现出了不同的分离选择性。

**图 6:**
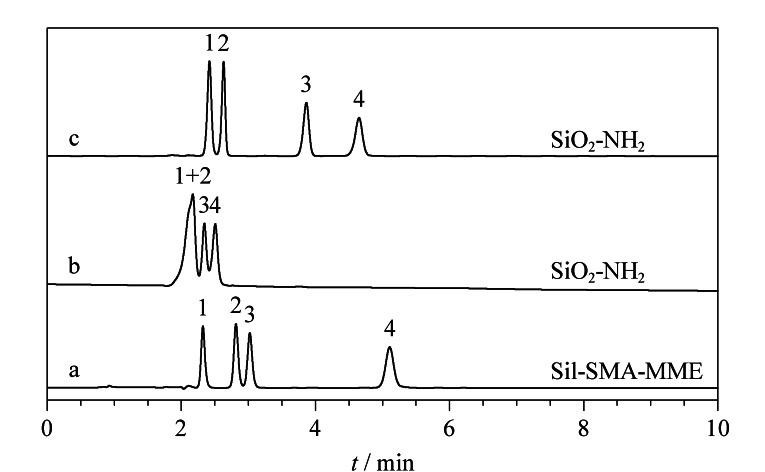
苯酚类物质在(a)Sil-SMA-MME色谱柱和(b, c)SiO_2_-NH_2_柱中的分离色谱图

### 2.4 Sil-SMA-MME色谱柱用于分离磷脂样品

#### 2.4.1 Sil-SMA-MME色谱柱用于分离磷脂标准品

基于Sil-SMA-MME色谱柱良好的分离选择性,进一步考察了Sil-SMA-MME色谱柱对磷脂的分离性能。首先选择不同类别的磷脂DPPS、DOPC和DPPE考察其分离选择性。如图7a所示,选用流动相为A-B(80∶20, v/v),其中,A相为正己烷-异丙醇-水-冰乙酸-三乙胺(5∶81∶14∶1.5∶0.08, v/v/v/v/v), B相为甲醇,3类磷脂DPPS、DOPC和DPPE可实现基线分离,且峰形良好。

**图 7:**
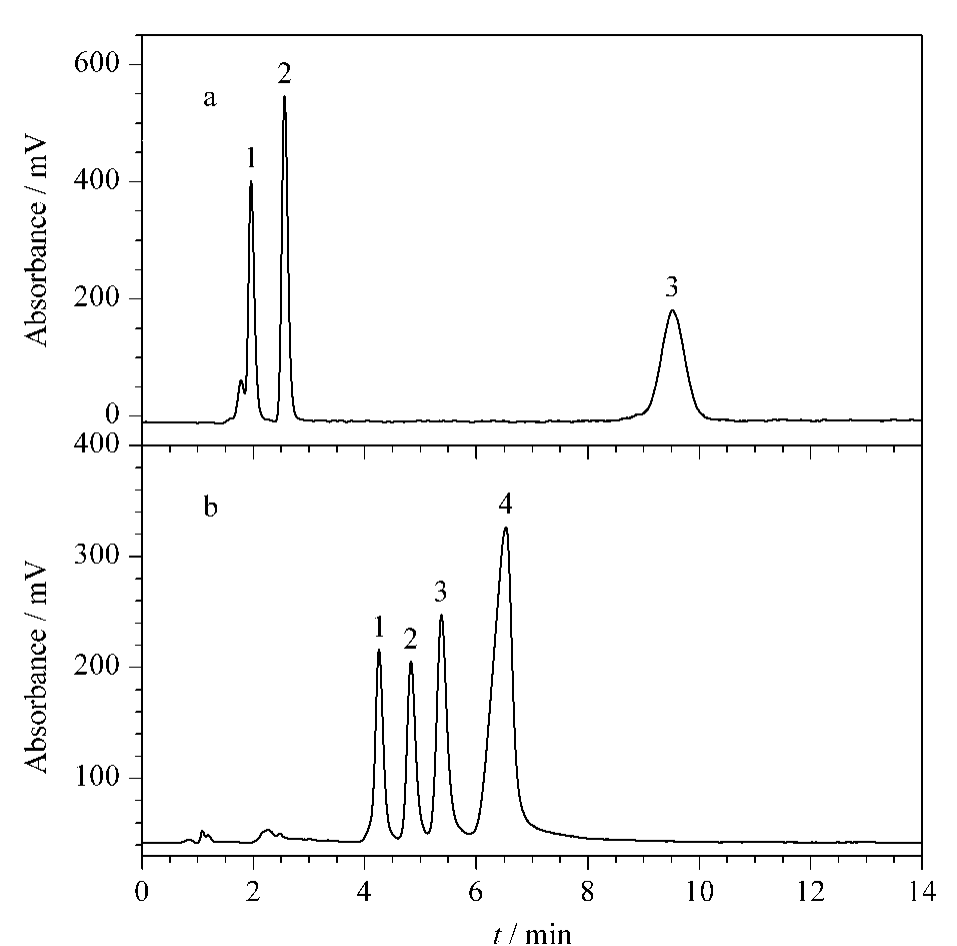
(a)不同磷脂类别和(b)不同PC分子种类在Sil-SMA-MME色谱柱中的分离色谱图

进一步采用不同的PC分子LysoPC、DMPC、DSPC和DPPC评价了Sil-SMA-MME色谱柱对不同种类磷脂的分离选择性。如图7b所示,选用流动相为A-B(25∶75, v/v),其中A相为水,B相为甲醇-乙腈(8∶1, v/v),具有不同酰基侧链的4种PC在10 min内实现了基线分离。以上结果显示,所制备的Sil-SMA-MME色谱柱可以同时实现磷脂类别和PC种类的分离分析。

#### 2.4.2 Sil-SMA-MME色谱柱用于分离复杂磷脂样品

南极磷虾油富含磷脂态的二十碳五烯酸(EPA)和二十二碳六烯酸(DHA)等Omega-3多不饱和脂肪酸,具有良好的生物活性,如调节血脂、缓解肥胖症并可提高人体免疫力和减轻脑老化等。磷虾油磷脂主要由PC、磷脂酰肌醇(PI)、磷脂酰乙醇胺(PE)、磷脂酰丝氨酸(PS)组成,其中PC为主要成分。采用南极磷虾油磷脂提取物考察了Sil-SMA-MME色谱柱对于复杂磷脂样品的分离性能。如图8a所示,选用流动相为A-B(25∶75, v/v),其中A相为水,B相为甲醇-乙腈(8∶1, v/v), Sil-SMA-MME色谱柱可以在10 min内分离出6个主要的色谱峰。

**图 8:**
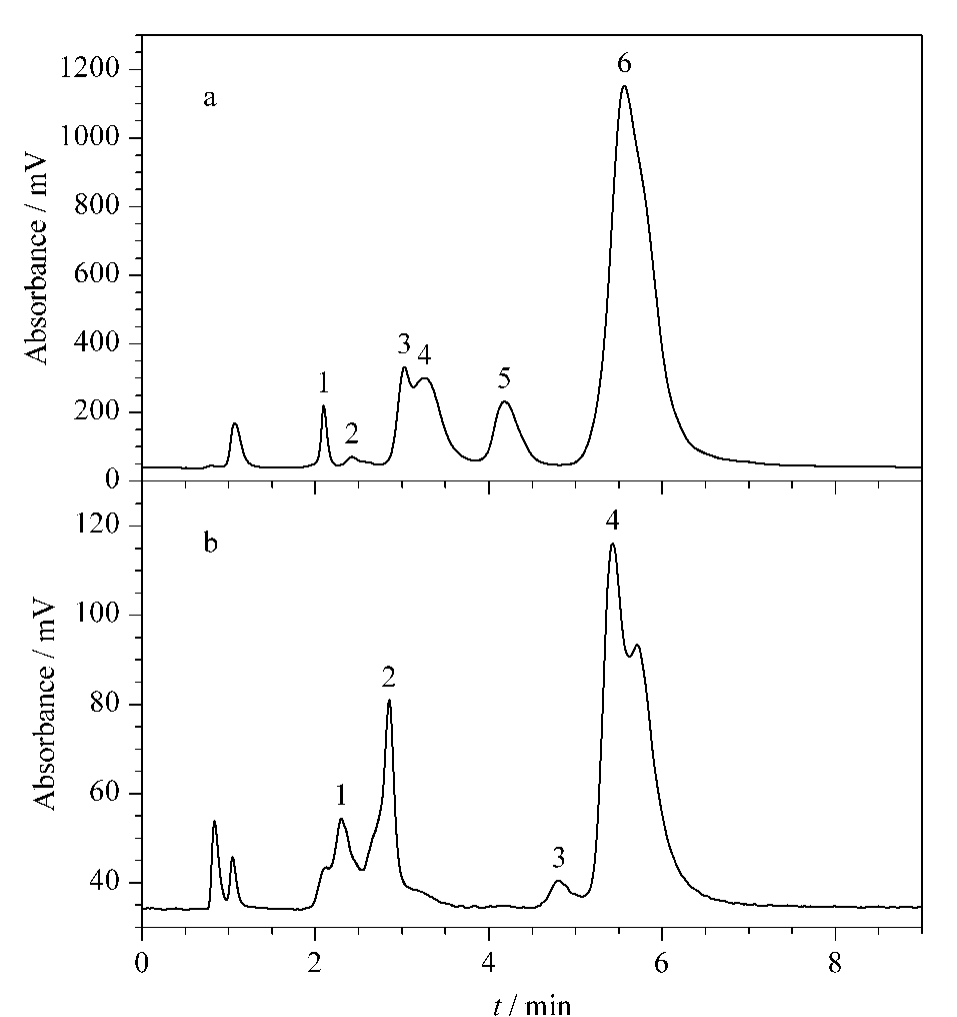
(a)南极磷虾油磷脂提取物和(b)人血清磷脂提取物在Sil-SMA-MME色谱柱中的分离色谱图

血清磷脂主要包括4部分——卵磷脂、溶血磷脂、神经磷脂和脑磷脂。血清磷脂的组成发生变化会导致多种心血管类疾病的发生,例如肥胖代谢综合征和糖尿病。另外,心力衰竭、类风湿性关节炎、骨质疏松症等疾病的发生也与体内磷脂的代谢密切相关。基于此,进一步采用人血清磷脂提取物考察了Sil-SMA-MME色谱柱对复杂磷脂样品的分离性能。如图8b所示,选用流动相为A-B(25∶75, v/v),其中A相为水,B相为甲醇-乙腈(8∶1, v/v), 10 min内可以观察到4个主要色谱峰。显然,Sil-SMA-MME色谱柱对复杂的磷脂样品显示了一定的分离潜能。

## 3 结论

本研究以苯乙烯和马来酸酐为单体,进一步结合MME·HCl开环修饰,制备了Sil-SMA-MME色谱固定相。填充Sil-SMA-MME色谱柱具有典型的亲水作用色谱保留机制,并且对于酰胺类和核苷/核酸碱基类以及苯酚类小分子物质显示了良好的分离选择性。此外,通过调节不同的色谱分离条件,Sil-SMA-MME色谱柱可以实现不同磷脂类别和PC种类的有效分离,并且对于复杂的南极磷虾油磷脂和人血清磷脂样品显示了一定的分离潜力。本研究为开发新型磷脂分离色谱固定相材料提供了新的可行性思路。

## References

[1] Nie J, Yang J, Wei Y, et al. Mol Aspects Med, 2020, 76: 100909 33023753 10.1016/j.mam.2020.100909

[2] Sun C, Ma C, Li L, et al. Chin Chem Lett, 2022, 33(4): 2073

[3] Das B, Nayak A K, Mallick S. J Drug Delivery Sci Technol, 2022, 76: 103780

[4] Correia A C, Monteiro A R, Silva R, et al. Adv Drug Deliv Rev, 2022, 189: 114485 35970274 10.1016/j.addr.2022.114485

[5] Kalvodová A, Zbytovská J. Int J Pharm, 2022, 628: 122264 36209979 10.1016/j.ijpharm.2022.122264

[6] Nasrollahzadeh M, Ganji F, Taghizadeh S M, et al. J Biosci Bioeng, 2022, 134(5): 471 36151004 10.1016/j.jbiosc.2022.08.003

[7] Magar K T, Boafo G F, Li X, et al. Chin Chem Lett, 2022, 33(2): 587

[8] Dong J, Ye F, Lin J, et al. Mitochondrial Commun, 2023, 1: 2

[9] Morita S-Y, Ikeda Y. Biochem Pharmacol, 2022, 206: 115296 36241095 10.1016/j.bcp.2022.115296

[10] Liu Y, Wu Y, Jiang M. Front Physiol, 2022, 13: 935195 35957983 10.3389/fphys.2022.935195 PMC9360546

[11] Wang X, Han X, Powell C A. Clin Transl Med, 2022, 12(5): e863 35588460 10.1002/ctm2.863 PMC9119606

[12] Kiddane A T, Roy V C, Kang M-J, et al. Chem Biol Drug Des, 2022, 10.1111/cbdd.14165 36303298

[13] Chang W, Xiao D, Fang X, et al. Cytotherapy, 2022, 24(2): 93 34742629 10.1016/j.jcyt.2021.09.013

[14] Grey M, Dunning C J, Gaspar R, et al. J Biol Chem, 2015, 290(5): 2969 25425650 10.1074/jbc.M114.585703 PMC4317028

[15] Vosse C, Wienken C, Cadenas C, et al. J Chromatogr A, 2018, 1565: 105 29983166 10.1016/j.chroma.2018.06.039

[16] Ansar S M, Jiang W, Mudalige T. J Pharm Biomed Anal, 2022, 208: 114473 34814079 10.1016/j.jpba.2021.114473

[17] Sun Y, Tian S, Hussain M, et al. Food Res Int, 2022, 161: 111872 36192991 10.1016/j.foodres.2022.111872

[18] Anesi A, Guella G. J Chromatogr A, 2015, 1384: 44 25655586 10.1016/j.chroma.2015.01.035

[19] Li L, Foley J P, Helmy R. J Chromatogr A, 2019, 1601: 145 31072602 10.1016/j.chroma.2019.04.061

[20] Hamimi S A, Sandahl M, Armeni M, et al. J Chromatogr A, 2018, 1548: 76 29567363 10.1016/j.chroma.2018.03.024

[21] Zhu C, Dane A, Spijksma G, et al. J Chromatogr A, 2012, 1220: 26 22169191 10.1016/j.chroma.2011.11.034

[22] Liang M, Liu D, Nie Y, et al. Chin Chem Lett, 2022, 33(6): 3123

[23] Zeng L, Jiang L J, Yao X D, et al. Chinese Journal of Chromatography, 2022, 40(6): 547 35616200 10.3724/SP.J.1123.2021.12023 PMC9404126

[24] Liu D, Wang H, Liang M, et al. J Chromatogr A, 2021, 1660: 462676 34814089 10.1016/j.chroma.2021.462676

[25] Li X T, Liang P, Zhou Y F, et al. Chinese Journal of Chromatography, 2020, 38(11): 1263 34213096 10.3724/SP.J.1123.2020.02012

[26] Cunningham R D, Kopf A H, Elenbaas B O W, et al. Biomacromolecules, 2020, 21(8): 3287 32672942 10.1021/acs.biomac.0c00736

[27] Esmaili M, Tancowny B P, Wang X, et al. J Biol Chem, 2020, 295(25): 8460 32358064 10.1074/jbc.RA119.012348 PMC7307199

[28] Harding B D, Dixit G, Burridge K M, et al. Chem Phys Lipids, 2019, 218: 65 30528635 10.1016/j.chemphyslip.2018.12.002 PMC6349512

[29] Neville G M, Edler K J, Price G J. Nanoscale, 2022, 14(15): 5689 35315461 10.1039/d1nr07230g

